# A qualitative study of perceptions of control over potential causes of death and the sources of information that inform perceptions of risk

**DOI:** 10.1080/21642850.2022.2104284

**Published:** 2022-07-29

**Authors:** Richard Brown, Elizabeth Sillence, Gillian Pepper

**Affiliations:** Department of Psychology, Northumbria University, Newcastle, UK

**Keywords:** Risk perceptions, health behaviours, information seeking, causes of death, health and lifestyle

## Abstract

**Background:**

Investigating perceptions of control over mortality risk may be fundamental to understanding health behaviours and tackling socioeconomic gradients in health. Few studies have explored perceptions of control over different causes of death and there is a lack of qualitative risk research. Our aim was to examine participants’ perceptions of control over potential causes of death and the sources that inform perceptions of risk.

**Method:**

We conducted semi-structured interviews with 24 participants (14 female and 10 male) and conducted a template analysis to analyse the transcripts.

**Findings:**

We identified six themes to represent participants’ perceptions of control over potential mortality risks and the sources that inform these perceptions: *Health-Related Mortality Risks, External Causes of Risk, Finding Balance, Family Medical History, Online Sources of Risk and Health-Related Information, and Health Misinformation*. Dying from heart disease was broadly reported as being a controllable risk, whereas cancer was mostly discussed as uncontrollable. Gender-specific cancers were perceived as posing a significant risk to life, however controlling this risk was discussed in terms of screening and treatment, not prevention. Family medical history was discussed as an informative source for longevity predictions, but less so for specific causes of death. Most risk information is retrieved from ‘Dr Google’, though trusted sources, such as NHS websites, are used for validation. Health misinformation online was seen as a problem experienced by other people, rather than the individual.

**Conclusions:**

Causal pathways between behaviours and specific cancers may not be obvious to individuals. Messages emphasising the broader links between diet, alcohol and general cancer risk may highlight the controllability of cancer risk through improved health behaviours. Furthermore, given the rise in health misinformation, and the belief that it is other people not ourselves that are typically susceptible to believing misinformation online, further attempts are needed to combat this growing ‘infodemic’.

## Introduction

Non-communicable diseases (NCDs) are responsible for 71% of annual global deaths, equalling over 40 million fatalities each year (World Health Organization, 2021b). Many types of NCDs (such as cardiovascular disease, diabetes and multiple forms of cancer) are heavily linked to health behaviours and are therefore often referred to as ‘lifestyle diseases’ (Tabish, 2017). A large portion of premature deaths could be avoided by targeting modifiable health behaviours (such as tobacco use, alcohol consumption, dietary intake, and physical activity) associated with prevalent NCDs (Yusuf et al., 2020). Investigating perceptions of control over mortality risk may help to inform health interventions aimed at improving health behaviours associated with lifestyle diseases.

Perceived uncontrollable mortality risk is that portion of risk of death which the individual believes cannot be mitigated by health effort (Brown, Coventry, & Pepper, 2021a; Nettle, 2010; Pepper & Nettle, 2014a, 2014b, 2017a, 2017b). The Uncontrollable Mortality Risk Hypothesis suggests that people who are more likely to die as a result of factors beyond their control should be less motivated to engage in positive health behaviours (Pepper & Nettle, 2014a). This is based on a behavioural ecological model for explaining social gradients in preventative health behaviours that states that the optimal individual investment in health behaviour should be less for people of lower socioeconomic status (Nettle, 2010). This is because people in lower socioeconomic positions typically experience greater exposure to environmental risks that are harmful to their health (Bolte, Tamburlini, & Kohlhuber, 2010; Cifuentes, Rodriguez-Villamizar, Rojas-Botero, Alvarez-Moreno, & Fernández-Niño, 2021; Evans & Kim, 2010; Fairburn, Schüle, Dreger, Karla Hilz, & Bolte, 2019). Pepper and Nettle (2014b) found that the effect of lower socioeconomic status on reported health effort was entirely mediated by perceived uncontrollable mortality risk. Additionally, recent research during the COVID-19 pandemic found that perceived uncontrollable mortality risk was associated with lower reported adherence to Government advice on diet and physical activity, as well as higher levels of smoking (Brown et al., 2021a). Therefore, investigating perceptions of control over mortality risk may be fundamental to understanding health behaviours and tackling socioeconomic gradients in health. Greater levels of perceived uncontrollable mortality risk have been associated with lower reported health effort in previous research, however the factors that are driving these perceptions of control are currently unknown (Brown et al., 2021a; Pepper & Nettle, 2014a, 2014b).

Few studies have explored perceptions of control over different causes of death. Wang et al. (2009) conducted a survey of perceptions of risk, worry, severity and control associated with heart disease, diabetes, stroke, and colon, breast, and ovarian cancers. Perceived control ratings were found to be highly variable across diseases with participants perceiving greater control over non-cancer conditions (such as heart disease) compared to cancers. However, our understanding of beliefs about control over different causes of death remains understudied, and little is known about what underpins and informs these perceptions of risk. For example, is risk of death due to cardiovascular disease perceived as being more controllable than all cancer-based mortality risk? If so, why? Given the relationship between perceived uncontrollable mortality risk and health behaviours, it is important to explore the specific beliefs that may be driving overall perceptions of uncontrollable mortality risk. Identifying whether some types of mortality risk are widely perceived as less controllable than others may help us to understand the relationships between sources of risk perceptions and subsequent health behaviours. For example, those who believe their risk of dying from cancer is beyond their control due to family medical history, may be less motivated to engage in those positive health behaviours likely to reduce their risk because they consider the risks to be unavoidable. In such cases, interventions tailored to emphasise the extent to which existing cancer risk is modifiable through positive health behaviours may prove effective. Furthermore, identifying those causes of death which are commonly perceived as being uncontrollable may help to identify actionable targets for structural and environmental interventions that would be most likely to generate improved health behaviours.

In order to understand perceptions of mortality risk we must consider where these perceptions come from. For example, in a survey of US senior citizens, perceived longevity prospects were consistently more responsive to precise information (age of parental death) than imprecise information (age of parent-in-law's death)(Chen, 2013). However, little is known about other sources that inform perceptions of uncontrollable mortality risk and what may determine the weight assigned to competing sources of information. For example, the extent to which someone believes they can control their risk of dying from a specific cause may be informed by individual consultations with healthcare professionals and other formal sources of information delivered by their healthcare provider. Alternatively, perceptions of control may be influenced more by one’s own family medical history and the personal experiences of health and risk reported within one’s social circle. The value that an individual assigns to either of these sources may contribute towards their overall beliefs about risk and influence their subsequent health behaviours. Identifying the sources of information that people use to assess their personal risk of death may help to capture the range of cues that inform perceived uncontrollable mortality risk (Brown, Coventry, & Pepper, 2021b). Understanding which sources carry the most weight will provide insights necessary for delivering interventions to influence health behaviours. Additionally, perceptions of risk can be strongly influenced by a variety of media factors. The amount of media coverage that a risk receives and the tone in which the information is presented can increase the perceived salience of a specific category of risk (Paek & Hove, 2017). The news media have been reported to significantly misrepresent the prevalence of leading causes of death which may lead to distorted perceptions of risk among the public (Frost, Frank, & Maibach, 1997). For example, heart disease accounts for close to one third of all US deaths, yet represents only 2–3% of media reports on mortality risks (Ritchie & Roser, 2018). Conversely, violent deaths (including suicide, homicide and terrorism) account for less than 3% of annual US deaths, yet represent more than two thirds of media coverage concerning risk of death (Ritchie & Roser, 2018). In addition to using traditional media as a source of information about risk, people are increasingly turning to self-help sources and consumer health informatics to learn more about their health (Zhao & Zhang, 2017). The growing presence that these forms of information have on social media is accompanied by rising concerns about health misinformation online (Suarez-Lledo & Alvarez-Galvez, 2021; Waszak, Kasprzycka-Waszak, & Kubanek, 2018). As the media landscape continues to diversify and social media plays a more dominant role, future research must play close attention to the influence that media coverage and related informational sources have on risk perceptions (Paek & Hove, 2017).

A lack of qualitative risk research has meant that some empirical studies have been led by ungrounded theoretical assumptions about how perceptions of risk are formed (Hawkes & Rowe, 2008). Qualitative research can help to map the domain of perceived control over mortality risk, which may serve as a guide for future quantitative studies used to survey the risk beliefs of broader populations. In depth qualitative study may also help to bridge between the quantitative comparison of perceived levels of risk across multiple diseases, and the study of information-seeking behaviours to investigate how risk perceptions are formed and develop. This may help to determine the extent to which individual beliefs influence how (qualitatively and quantitatively) risks are perceived (Hawkes & Rowe, 2008). Therefore, we conducted a qualitative study of perceptions of control over mortality risk using semi-structured interviews. The aim of this study was to explore participant beliefs about control over potential categories of mortality risk and to identify the informational sources that inform these beliefs. The primary research questions were (1) *How do beliefs about control vary among different causes of death?,* and (2) *How do people use different sources of information to inform their beliefs about control over mortality risks?*

## Methods

### Sample and recruitment

This study was approved by the Department of Psychology Ethics Committee (33427) at Northumbria University. Our full study protocol is registered with the Open Science Framework (osf.io/eyhfu/)*.* Participants were recruited by approaching Facebook community pages local to Newcastle Upon Tyne. Community pages representing eight local areas were selected with the aim of recruiting participants from a diverse range of socioeconomic backgrounds. This sampling method addresses concerns raised by a systematic review of risk research which highlighted that the predominant use of convenience samples in previous research has failed to reflect the importance of socioeconomic factors in studying risk perceptions (Hawkes & Rowe, 2008). Newcastle is a suitable city for recruiting participants from varying socioeconomic positions because it covers a broad range of levels of deprivation within a relatively small geographic space (Ministry of Housing, 2019b). Participants completed an initial screening survey to provide basic demographic information including age, sex, gender, education, income and postcode. There were no restrictions on gender, sexual orientation or upper age limit, but participants were required to be aged 18+ and currently residing in Newcastle Upon Tyne. The sample size was chosen in consideration of a systematic review which found that the majority of successful qualitative interview studies of risk perception recruit samples of 20–50 participants (Hawkes & Rowe, 2008). However, more recent guidance on determining sample sizes in qualitative research suggests that samples approaching and exceeding 30 participants can become unwieldy to administer and analyse (Boddy, 2016). Therefore the study recruited 24 participants (14 female and 10 male). The mean age of participants was 40.1 years (ranging from 21 to 67). Participant postcodes were used to generate deprivation scores based on the ONS’ Indices of Multiple Deprivation (IMD) (McLennan et al., 2019; Ministry of Housing, 2019a), which we used as an indicator of participant socioeconomic status. Our iterative sampling process consisted of inspecting the IMD scores of recruited participants (after approximately one quarter, one half, and three quarters of our target sample size had been recruited) and subsequently reposting study advertisements to those Facebook community pages representing areas known to have higher/lower than average levels of affluence (depending on the recruited sample’s average IMD score). This allowed us to ensure that participants were recruited from a broad range of socioeconomic backgrounds. The IMD decile scores for our sample ranged from one (most deprived) to ten (least deprived) with the mean deprivation score falling within the sixth decile.

### Data collection

All participants took part in semi-structured interviews, conducted using an interview schedule designed to explore participants’ beliefs about the controllability of different causes of death and to identify the informational sources and behaviours associated with personal mortality risk (for full interview schedule see preregistration document; osf.io/eyhfu/). Participants were first asked to provide a measure of perceived uncontrollable mortality risk by stating a score for their believed likelihood of living to 81 (current average UK life expectancy) provided they make the maximum effort to look after their health (Pepper & Nettle, 2014b). With 0 representing ‘no chance’ and 100 representing ‘certain’, the reported score is deducted from 100 to ascertain the portion of mortality risk that is believed to be beyond their control. This measure was used to prompt participants to consider their degree of control over the risks to their long-term health and safety, and to ask participants to reflect on their anticipated longevity (participant scores are provided in the Supplement, see Table S1). Participants were then asked to describe how they arrived at their chosen response in order to discuss factors that influence their calculation of personal risk. Participants were then asked to discuss causes of death that come to mind when considering their own personal risk of death. Participants were free to consider all potential risks that they felt could be categorised as a potential cause of death. Participants were prompted by reference to the most common categories of avoidable death in the UK (Office for National Statistics, 2021) and most prevalent public risks identified by the National Risk Register (Cabinet Office, 2020)(for a full list of prompt categories of mortality risk, see Supplement Item S1). The interview schedule was collectively agreed upon by the full research team and was consistently applied by the same researcher. The interviewer asked participants about information seeking and avoidance behaviours relevant to personal risk of death and the sources of information that inform their perceptions of control over mortality risk. Interviews were conducted via Microsoft Teams to ensure that participants were not required to download or install any software to their chosen device. Interviews lasted between 24 and 56 minutes with an average interview time of 36 minutes. Interviews were digitally recorded and subsequently transcribed verbatim. During the transcription process, all identifying information was pseudonymised with participants subsequently being referred to by participant number.

### Data analysis

The participants’ responses were studied by conducting a Template Analysis of the transcripts, a method which allows the researcher to identify and define *a priori* themes that depict topics and concepts that are of interest and relevance to the study (Brooks, McCluskey, Turley, & King, 2015; King, 2012). This analytic approach allowed for the findings to focus on beliefs and perceptions of control relevant to those categories of mortality risk and avoidable death most prevalent in the UK (Cabinet Office, 2020; Office for National Statistics, 2021), whilst still allowing for the addition of further themes to reflect participants’ responses. This approach also meant that the findings from participants’ discussions of informational sources considered both those sources identified by previous research as being central to health-information seeking, as well as more novel cues of health and risk. The process of developing the initial template involved reviewing the content of each transcript before conducting a preliminary thematic coding of the transcripts in alignment with *a priori* themes. This coding process was conducted using NVivo 12. Clusters of additional themes and topics of interest were then analysed to produce superordinate themes and combined to produce the initial template. This was then applied to the interview data and subsequently modified to consolidate the themes and structure. The final version of the coding template was agreed upon by all research team members and led to the selection of six themes representing the participants’ perceptions of control over different mortality risks and the informational sources and behaviours that inform these perceptions. Verbatim extracts from the transcripts are presented and discussed below to illustrate the findings.

## Findings

We report the findings of our interview study by presenting the themes that were highlighted from our template analysis as best representing the transcripts (see Table 1). These themes are divided by research question to clearly present how the findings of this research address the directives of the study. An overview of each theme, accompanied by representative quotations from the transcripts, are presented in Table 1. Each theme is presented, defined and explored in detail below.

**Table 1.** Definition of themes and example quotations.

Theme title	Definition	Example quotations
*Research Question 1: How do beliefs about control vary among different causes of death?*		
Health-related mortality risks	Increased risk of dying from heart disease is believed to result from health and lifestyle factors, whereas dying due to cancer risk is perceived as being more uncontrollable than controllable. Female participants are most concerned about developing and dying from female-specific cancers, and are focussed on screening and treatment, as opposed to prevention.	*‘I think it would probably be more cardiovascular. I mean I suppose there’s cancer as well, but I think everyone thinks heart attack when they see chips!’ [Participant 19] ‘I'm not sure, but there’s definitely a feeling of a raffle kind of thing going on where you pick a number and you might get it, certain cancers, not all cancers.’ [Participant 12]*
External causes of risk	Multiple external risks are believed to pose a threat to life. Traffic accidents and the effects of pollution are discussed as uncontrollable risks with a high level of uncertainty, whereas the risks from violence and accidents in the home are believed to be more manageable.	*‘You will always get those random car accidents that happen that you can't control.’ [Participant 11] ‘It's not something that I dwell on, but I think that's partly because there isn't a lot that you can do [when driving] other than just, you know, do your best.’ [Participant 7]*
Finding balance	Participants balance their investment in a healthy lifestyle and the mitigation of external risks against competing interests. For example, personal coping strategies for managing daily stress (i.e. comfort food) may take preference over a healthy lifestyle. This is also true of balancing the perceived convenience of driving at high speeds against the increased exposure to mortality risk.	*‘You've gotta decide whether your comfort levels, like the emotional support you get from whatever the habit is … like whether the risk outweighs the day-to-day experience.’ [Participant 10] ‘It's the last thing I want to do on a night time, prepare a really fresh meal and then go for a two mile jog … I want to sit in front of the TV. So I think sometimes like the busy, busy lifestyle definitely gets in the way of being as healthy as I'd like to be.’ [Participant 16]*
*Research Question 2: How do people use different sources of information to inform their beliefs about control over mortality risks?*		
Family medical history	Family medical history informs perceptions of control over mortality risk but centres on anticipated longevity rather than predispositions to specific risks.	*‘My mom's mom, died at the age of [age]. So in my head it's always been, I need to get past [age]. I need to get past [age] because then I'm OK and it's always been a bit like a number that's been in my head … like, well, she died young so I could too.’ [Participant 24]*
Online sources of risk and health-related information	The internet is the most commonly used source of risk and health-related risk information. Participants broadly consult ‘*Dr Google*’. Some participants then ascertain ‘Trusted Sources’ to validate the information received, whereas others do not discern between search results and consider ‘*Dr Google*’ to be their primary source.	*‘If I was worried or feel something, you kind of have that Google thing, don't you? You try to become your own doctor and, and you’re looking up things.’ [Participant 18]*
Health misinformation	Health misinformation is believed to be prevalent on social media. People generally believe that they are not susceptible to online health misinformation, but that others are.	*‘I would say definitely not with my close social circle or myself, but definitely kind of acquaintances and colleagues. People like that who I'm not necessarily kind of that close to have definitely seen stuff where, like I was gonna say fake news, but you know, kind of whatever and it is basically changing people's opinions on stuff.’ [Participant 15]*

## RQ1: How do beliefs about control vary among different causes of death?

Participants reported a range of beliefs about how much individual control they feel they have over potential causes of death. Clear trends were presented with respect to control over different causes of death due to health risks, external causes of risk, and the need to balance the pursuit of a healthy lifestyle and avoidance of risk against other competing interests. For example, though dying from heart disease was widely discussed as a controllable risk, cancer-related death was mostly reported as being beyond individual control. External causes of death such as traffic accidents and environmental pollution were mostly seen as uncontrollable risks. Finally, participants described how making healthy decisions and avoiding external causes of risk can often compete with the demands of a busy schedule, coping strategies for dealing with stress, and the convenience of accepting some risks.

### Health-related mortality risks

Heart disease was exclusively discussed as a potential cause of death that could be controlled through health behaviours. Roughly half of participants (11/24) specifically linked health and lifestyle factors to risk of heart disease.

I suppose lots of things to do with cardiovascular are related to healthy eating and diet and exercise, so I think that's probably the thing that I feel you have the most control over. Other things, just luck, I think. [Participant 14]

If you've got saturated fats, you’ve got high levels of cholesterol. They are going to impact your arteries and they’re gonna block them up. This is all like science. That will lead to heart disease. [Participant 20]

I think it would probably be more cardiovascular. I mean I suppose there’s cancer as well, but I think everyone thinks heart attack when they see chips! [Participant 19]

Over half of participants discussed their risk of dying from cancer as being something that is mostly uncontrollable.

I'm not sure, but there’s definitely a feeling of a raffle kind of thing going on where you pick a number and you might get it, certain cancers, not all cancers. [Participant 12]

Four participants suggested that some cancer risk can be avoided, though this was predominantly discussed in reference to lung cancer being avoided by not smoking. Mostly, the perceived uncontrollability of cancer risk was supported by reference to reports of people who have ‘done everything right’ from a health perspective and have still become ill or have died from cancer.

I know friends and, kind of, friends of friends who've developed forms of cancer and haven't done any of those things [negative health behaviours] and have otherwise been healthy, so it feels like there are definitely things that I can do, but there are forms of cancer that that you might get irrespective of doing those things. [Participant 6]

They always talk about, you know, eat some fruit and vegetables you know, should help reduce your risk of cancer. But, there is always, you know, equally the most fit person in the world can get cancer as well. [Participant 11]

Approximately half of all female participants (6/14) explicitly expressed concerns about their risk of developing and dying from female-specific cancers (i.e. cervical, ovarian and breast cancers). In contrast, no male participants expressed concerns about male-specific cancer risk (such as prostate, penile and testicular cancers).

I don't smoke, I feel like my risk of getting lung cancer would be sort of slim, but other cancers … I do worry about breast cancer, ovarian cancer, and more sort of female related cancers. [Participant 16]

When you think of those more specific cancers, I think it's, it's difficult. Female cancers like breast cancer. I'm not sure what you can do to control that. [Participant 19]

When addressing what can be done to control mortality risk from female cancer, discussions focussed on screening and treatment, rather than prevention and health behaviours.

My control over that is going for, you know, smear tests at the doctors as soon as I'm invited … but in terms of … you know, catching an illness … I feel like there's little control. [Participant 16]

So it’s whether you keep up with your smears and you engage with those programs, because they are there, you know, that's your responsibility because it is proven that that will help, right? [Participant 24]

There was no discussion of health and lifestyle or external causal factors with respect to specific cancer risks. This relates to the reported lack of certainty regarding the link between health behaviours and increased risk from specific cancers, other than the frequently discussed impact of lung cancer on smoking.

The act of smoking is very definitely a breathing thing, so you would expect there to be lung problems. It's less obvious to me, what would be related to an instance of cervical cancer. [Participant 2]

### External causes of risk

Traffic accidents were the most commonly cited category of external risk of death and were discussed by the majority of participants (18/24). More than half of all participants suggested that traffic accidents pose the greatest external risk to their life.

I think the biggest risks for me. Yeah, around traffic, things like that. I think that's what I worry about the most. Probably that is my biggest sense of risk. [Participant 14]

I think every day I encounter somebody who you know put … if I didn't react carefully you know someone who would potentially, put my life at risk on the roads. [Participant 3]

Despite participants pointing to some elements of road safety that can reduce their exposure to risk, all participants that discussed risk of harm from traffic accidents highlighted that there would always be a significant portion of risk that is beyond their control.

I think that just as a driver, you're always weighing up risks you’re always trying to anticipate where this person's coming from, from what are they doing? And some of that you do control, but actually realistically a lot of it isn't really. You will always get those random car accidents that happen that you can't control. [Participant 11]

It's not something that I dwell on, but I think that's partly because there isn't a lot that you can do other than just, you know, do your best. [Participant 7]

One third of participants (8/24) discussed accidents in the home as a category of external risk that could potentially lead to serious injury or death. The most frequently mentioned types of accidents in the home were risks from accidental fires (three participants) and falling (four participants).

The main like fear that I have is like of … oh this sounds ridiculous, I'm aware … is of like falling, so like falling down the stairs … That’s like, part of like your everyday life. So like walking down the stairs like, when I walk down the stairs, I think I could fall to my death down here. [Participant 15]

It's a bit like final destination. You kind of think. Well, he [family member] went that, so I'll go that way. And that hasn't happened today because I just tend to avoid, you know, doing anything risky in that way, so I don't. I don't have a, we don't have a gas hob, we don't cook with oils like that. All these things I do to try and avoid that fire risk. [Participant 5]

Some participants discussed actions that can be undertaken to mitigate risks at home but there was still a sense that such risks are a common feature of everyday life. Participants highlighted that these risks were often at the forefront of their concerns about mortality risk despite believing that they may appear to be irrational concerns to others.

Overall, participants indicated that they felt the level of risk to their life from unavoidable violent attack was very low. However, three quarters of participants (18/24) did highlight that there are a number precautions and protective strategies that can be implemented to mitigate the risk of violence.

I don't enjoy feeling like I need to take particular precautions to go out and be in the world by myself, but I do feel like it's necessary. And to not do it … if something did happen, then I'm sure I would feel more culpable … you then beat yourself up with guilt for having not avoided it in the first place. [Participant 2]

When asked to consider external risks to health from their surrounding environment, just over half of all participants (13/24) discussed pollution as presenting a potential risk. Pollution was mostly discussed in terms of air and water pollution. Though participants believed pollution was a concern, they generally reported not having previously thought about the direct effect that pollution could have on their health.

I've always, you know, used the water supply to the house freely. I've never thought about that at all. I think very more recently I'd say air pollution is in the media a lot more. It's in the sort of local consultations … But I’ve not thought about it in terms of health, not to the extent that it's risking my health. [Participant 16]

The problems posed by pollution were also generally thought of as something that was beyond individual control.

We don't really consider it [pollution] because there's nothing I can personally do about it … You know these risks that are too, too big for one person to try and mitigate against, and I think, you know, that’s things like pollution. Yes, you know we do our bit. We try to use the cars less, we recycle, we do all of those things, but actually, you know, I can't stop the traffic. [Participant 3]

### Finding balance

All participants highlighted lifestyle decisions as being central to long-term health and mitigating mortality risks. Pursuing a healthy lifestyle and avoiding risk can sometimes be overshadowed by the demands of a busy schedule, personal strategies for managing stress and the convenience and rewards of accepting some risks.

Participants indicated that lifestyle behaviours were the most impactful factors on long-term health and risk of death, and also reported believing they have a high degree of control over what happens to their body.

I think just mainly lifestyle and exercise, smoking, your diet, that sort of thing that is under your control, what you actually do to your own body. [Participant 11]

So my diet … so I feel like I'm in control of my diet and I think that's probably the biggest risk factor. [Participant 12]

I think lifestyle, food is the biggest controllable risk that you can have. That's the one that you have most control over. What goes in your body. [Participant 4]

Despite reporting a broadly held sense of control over individual lifestyle factors, all participants indicated that the extent to which they follow a healthy lifestyle could be improved, this was most reported with respect to diet.

I mean if a report that says eating salted caramel is really bad for you, like ‘salted caramel gives you cancer’ … Are you going to stop eating it? Absolutely not! So, like I said, I will take it on board, but you filter the vast majority of risk. [Participant 19]

It's the last thing I want to do on a night time, prepare a really fresh meal and then go for a two mile jog … I want to sit in front of the TV. So I think sometimes like the busy, busy lifestyle definitely gets in the way of being as healthy as I'd like to be. [Participant 16]

Participants described balancing the extent to which they pursue a healthy lifestyle, and their acceptance of certain mortality risks, with competing interests they experience on a day-to-day basis. The most frequently mentioned competing interest was in reference to strategies that participants use to manage stress (i.e. consuming alcohol and unhealthy foods; discussed by 8/24 participants) which may provide short-term comfort in response to daily stresses.

It’s hard to say ‘you know what … I’m in this really difficult place where I’m struggling to come to terms with everything mentally, what I really want to do is crack on with a diet!’ You know, the only thing you really want to do is reach for another chocolate bar and have a bit of comfort. You've gotta decide whether your comfort levels, like the emotional support you get from whatever the habit is … like whether the risk outweighs the day-to-day experience. [Participant 10]

In theory, it's very easy to suggest to yourself or other people that you eat differently, but when things are habitual and you rely on these things for like short term psychological advantage, you know, if you’re just trying to boost your mood to get yourself through a difficult day and stop yourself sliding into like chronic low mood or depression. You know sometimes a little boost in terms of morale … like, that can make a big difference. [Participant 7]

Finally, the extent to which participants reported being willing to make lifestyle decisions to avoid certain risks was balanced against the convenience and potential rewards offered by riskier alternatives.

I know there's a safer way that I could drive and probably avoid injury. I think I've kind of come to terms with that in my head. I'm not saying I'm a wild driver, but I know that I could drive safer … I want to get where I'm going relatively quickly. So I think, I think it's convenience that’s probably to blame. [Participant 5]

There’s the risk of being attacked in the street, but I really like going to the theatre so … walking from the car to the theatre, especially if you were walking by yourself … but I really want to go and see a play! So I would take a risk. [Participant 9]

## RQ2: How do people use different sources of information to inform their beliefs about control over mortality risks?

Most participants discussed personal sources of information, most notably their family medical history, as providing a key source of health and risk-related information. Family medical history was principally referred to as providing an informative source from which to predict personal longevity. Participants also highlighted the internet as being a central provider of risk-related information. Participants’ reports about the increasing use of the internet to inform about health and risk were accompanied by concerns about the growing problem of health misinformation online. This problem was recognised by our sample as having the potential to influence the health beliefs and behaviours of others, though most felt they would not be susceptible to believing inaccurate health messages themselves.

### Family medical history

Over one third of participants (9/24) explicitly referred to their family medical history as a source of information that they consulted when providing a measure of perceived uncontrollable mortality risk. Roughly one quarter of participants (7/24) referred to specific causes of death within their family and how this made them aware of the possibility of an increased predisposition to dying due to such risks. However, when reflecting on family medical history, participants mostly focussed on the age of death rather than the quality of life or state of health of their relatives, and some (4 participants) reported that familial age of death could become a personal fixation.

My mom's mom, died at the age of [age]. So in my head it's always been, I need to get past [age]. I need to get past [age] because then I'm OK and it's always been a bit like a number that's been in my head … like, well, she died young so I could too. [Participant 24]

I think for me that that kind of risk of family history is quite, it's quite a big factor. I heard something new today … I heard something on one of the telly programs … it was a GP surgery and they said something like they quite often get people coming in around about the same age that their parents died … Actually, as I'm getting towards that age, it is more on my mind. I could understand that. So … that's something that I, I do consider, quite, you know, quite often. [Participant 3]

### Online sources of risk and health-related information

The majority of participants (22/24) reported using the internet as a key source of information about risks to their health and safety. When asked for information about their primary source of health information online, several participants (7/24) expressly stated that they refer to Google for health and risk-related information. Additionally, some participants reported that they did not discern between the different potential sources of information produced by a Google search, suggesting that they considered Google itself to be the information source rather than the means through which different information sources are accessed.

If I was worried or feel something, you kind of have that Google thing, don't you? You try to become your own doctor and, and you’re looking up things. [Participant 18]

I’m known to be a bit of a Googler who looks at a couple of sources online. I mean, I don't just look at the first website and that, but if I have back pain or leg pain, and it's all of a sudden … I'll certainly sort of Google it. [Participant 16]

It usually goes as far as Dr Google. [Participant 4]

However, many participants were keen to highlight that after initially using google to search for information, they then go on to validate the information by consulting existing sources that they trust (i.e. NHS websites, charities, and known health organisations).

I always tend to look for the NHS ‘cause for me that's a reliable source of information. I think there's so much nonsense out there on the Internet that's so unreliable and absolutely ridiculous. Especially when it comes to treatment and things, you’ve got to be so careful. [Participant 11]

Like Macmillan Cancer and the Marie Curie. Those sort of recognized websites, so I think I deliberately choose ones that I think have some sort of recognized authority behind them. [Participant 14]

Most participants (17/24) indicated that they do not actively seek out information about risks to their life, unless prompted to do so by specific health incidents that they wish to understand in greater detail.

I would say I don't actively look for you know, info about risks to my health … If I’m feeling poorly, I go to the doctors … and maybe that leads me into researching a little bit more about what it is. [Participant 13]

I think. I think unless it applies to me specifically, I don't necessarily go out looking for it. So for example, you know, I'm not necessarily expecting to have a stroke or a heart attack, you know, so I don't necessarily go out looking for information about that. [Participant 3]

### Health misinformation

When asked about the presence of health-related misinformation online, over half of participants reported the belief that they regularly encounter health misinformation on social media. Participants indicated that they believed health misinformation relating to COVID-19 was most prevalent but that there is also a strong presence of more general health-related misinformation online. However, only one participant out of the 13 who discussed health misinformation on social media felt that they would be susceptible to believing inaccurate health messages online. Despite reporting that their own attitudes and behaviours are not affected by health misinformation, most participants believed that others are highly susceptible to believing inaccurate messages about health online.

I would say definitely not with my close social circle or myself, but definitely kind of acquaintances and colleagues. People like that who I'm not necessarily kind of that close to have definitely seen stuff where, like I was gonna say fake news, but you know, kind of whatever and it is basically changing people's opinions on stuff. [Participant 15]

So yeah, [health misinformation], it's a bit of a minefield, but I feel reasonably confident that I'm only retrieving accurate information. [Participant 2]

I guess it’s a snobbish attitude on my part … like, you know, it’s the same people sharing this stuff. The same conspiracy theory people are sharing this stuff. The same conspiracy theorists thought that we were sending 350 million a week to Europe. It's the same people over and over and over and over again that fall for all of this and it's really depressing that that happens. [Participant 5]

In addition to the sources of information described above, participants also discussed receiving information about risks to their long-term health and personal safety from personal experiences of health, healthcare professionals, self-monitoring health devices, TV and radio. These additional sources were captured by our initial template but are not presented as central findings due to redundancies among these themes (for full details of the initial template, see Supplement Item S2).

## Discussion

Participants discussed a broad range of both health-related and external mortality risks that they considered to pose a significant risk to their lives. The perceived controllability of these risks was described as existing on a continuum ranging from completely beyond individual control to 100% controllable. The positioning of each discussed category of risk along this continuum varied by participant. However, certain potential mortality risks were broadly discussed by our sample as being controllable/uncontrollable. The external risks to health posed by traffic accidents and air pollution were broadly believed to be beyond individual control. In terms of health-related mortality risks, dying from general cancer risk and female-specific cancers (such as cervical, ovarian and breast cancer) were largely thought of as uncontrollable risks. This was discussed in contrast to the risk of dying due to heart disease, which was strongly and consistently linked by our sample to health and lifestyle behaviours, most notably diet. We will discuss the implications that this has for targeting improved health behaviours associated with multiple types of cancer.

Though family medical history was the most broadly discussed source of information when predicting personal longevity, it was discussed less often with respect to personal awareness of increased risk from specific causes of death. The most commonly described source of information for risks to long-term health and safety was ‘*Dr Google*’ although participants generally described their approach to risk information-seeking as being reactive to health occurrences rather than proactive risk management. The implications of our findings and suggestions for future research are discussed below.

### Perceptions of control over different mortality risks

When discussing the perceived controllability of different health-related mortality risks, participants indicated that increased risk of dying due to heart disease is broadly believed to result from health and lifestyle factors, whereas cancer risk, in general, is believed to be more uncontrollable than controllable. This supports the findings of a previous quantitative health study comparing perceptions of risk and control across multiple diseases which found that heart disease was believed to be the most controllable compared to cancers, which were perceived to be the least controllable (Wang et al., 2009). This has been suggested to be linked to the perceived severity of these conditions, where heart disease is viewed as less severe and more controllable in comparison to cancer (Scheideler, Taber, Ferrer, Grenen, & Klein, 2017). With respect to specific types of cancer risk, participants from our sample frequently discussed the known effects of smoking on increasing lung cancer risk, often dismissing the risk of dying from lung cancer as highly avoidable if they don’t smoke. However, discussions concerning other types of cancer, or cancer risk more broadly, did not address levels of engagement with personal actions that may reduce individual cancer risk. A recent review of cancer research suggested that, due to the fact that causal pathways for specific types of cancer can be hard to pin down, public health policies should focus on communicating the known major dietary risk factors associated with general cancer risk, particularly the effects of obesity and alcohol (Key et al., 2020). Emphasising the importance of diet and reducing alcohol consumption provide clear targets for behaviour change and increase perceptions of control, specifically where there may be high uncertainty concerning the causal factors associated with cancer risk.

Participants mostly discussed general cancer risk, as opposed to specific types of cancer, with the exception of lung cancer and ‘female-specific cancers’ (i.e. cervical, ovarian and breast cancers). Half of all female participants (7/14) were expressly concerned about developing and dying from female-specific cancers. This supports previous findings that women perceive themselves to be at greatest risk from breast and ovarian cancer compared to other equally prevalent health risks (Wang et al., 2009). In a quantitative survey of 439 women, heredity factors were perceived as being most impactful for breast cancer risk (Wang, Miller, Egleston, Hay, & Weinberg, 2010). This helps to explain our sample’s apparent focus on screening and treatment in response to the risks posed by female cancers as opposed to the dietary focus that was emphasised in response to the risks associated with heart disease. The discussed importance by our sample of engaging with early screening, detection and treatment programmes signals the success of efforts by public health communicators in raising the awareness of female-specific cancer. However, if the perceived levels of control in our sample are indicative of the perceptions of the broader UK population, our findings may suggest that further effort is needed to highlight the behavioural and lifestyle factors that may increase risk of developing and dying from female-specific cancers (Wang et al., 2009, 2010). Finally, it is also noteworthy that when discussing individual cancer risk, no male participants from our sample expressed concerns about their own risk of developing and dying from prostate cancer. Prostate cancer is the most common form of cancer among UK males (Cancer Research UK, 2021). This also highlights the need for future public health efforts to further emphasise the risks of prostate cancer to men and to communicate the personal strategies that may help to reduce levels of individual risk.

Traffic accidents were the most discussed category of external cause of death and discussions centred on the uncontrollability of their occurrence. Participants largely discussed the prevalence of accidents and inability to protect themselves from the actions of others, as opposed to the driving behaviours that they can practice in order to reduce their own level of risk. Despite the belief that driving poses a significant risk to life, only one participant referenced a personal experience as having informed this belief. Perceptions of risk and controllability may be influenced by longstanding campaigns to improve road safety. Previous research has suggested that where road safety advertising has focussed on eliciting fear by portraying violent car crashes, without providing statements of efficacy, drivers may not believe that their behaviours strongly influence the occurrence of crashes (Pedruzzi, Swinbourne, & Quirk, 2017). Though violent car crashes were previously the focus of UK road safety advertising for many years, these findings highlight the importance of the more recent focus on best practice driving behaviours to increase perceptions of control over the risks from driving (Department for Transport, 2021). Further research may look to investigate whether or not the perceptions of low controllability over traffic accidents reported by our sample are reflected in the broader UK population. Further study may also examine whether or not there is a delay between the enactment of changes in emphasis in road safety advertising and the resulting change to public perceptions about risk.

When asked about environmental mortality risks, half of all participants reported that pollution (most notably air pollution) posed a potential risk to life. However, most participants admitted that they do not ordinarily associate the effects of pollution with risks to their health. Participants also suggested that they tend not to dwell on the impacts of pollution to health as they ultimately feel little control over their exposure to air pollution. A review of qualitative research into health perceptions of air pollution since the 2000s found that the link between air pollution and human health is not always obvious from a lay perspective (Noël, Vanroelen, & Gadeyne, 2021). This may explain the broadly held awareness of air pollution but lack of direct concern for health, which is particularly apparent where individuals feel there is little they can do to mitigate the risk (Bickerstaff & Walker, 2001). If the wider UK population share the view depicted by our sample that little can be done to personally manage individual risk from air pollution, public health communication efforts may look to increase perceptions of control over this category of risk. Though policies aimed at reducing emissions at their source are clearly preferable, various types of individual action can be taken to reduce the risks associated with air pollution (Laumbach, Meng, & Kipen, 2015). For example, the risks from both ambient and household air pollution can be reduced by limiting physical activity outdoors during periods of high air pollution, reducing near-roadway exposure, wearing facemasks in prescribed circumstances, using air filters in the home and ventilating and isolating cooking areas (Carlsten, Salvi, Wong, & Chung, 2020). It is possible that highlighting the utility of these individual actions for reducing the harmful effects of air pollution may help to increase the level of perceived control over this category of mortality risk. However, it should be acknowledged that the option to reduce one’s exposure to pollutants may not be available to all. A global review of socioeconomic disparities in exposure to air pollution found that those of lower socioeconomic status in North America, Asia, Africa and other parts of the world experience higher concentrations of air pollutants (Hajat, Hsia, & O’Neill, 2015). In addition, income inequalities and additional socioeconomic factors may even aggravate the effects of pollution on health, creating an ‘environmental-health-poverty trap’ (Yang & Liu, 2018). Those with less access to resources may be unable to enact lifestyle changes to reduce the risks of air pollution (i.e. through purchasing home air filtering and ventilation systems, or choosing occupations and neighbourhoods with lower exposure to pollutants). Therefore, interventions aimed at encouraging individual action to reduce exposure to air pollution should be careful not to present solutions which may ultimately exacerbate existing health inequalities.

Perceptions of control over different causes of death varied by participant. However, believing that a particular mortality risk is generally controllable does not always translate into lifestyle decisions that actually reduce exposure to risk. Participants discussed a range of competing interests that might outweigh the decision to invest in behaviours to reduce your chances of mortality risk. Participants mostly discussed their awareness of how their approaches to managing stress may act against their desire to improve their health and avoid risk. This was most evident with respect to diet. This supports previous research from a study of over 4,000 UK residents in 2018 which found that 46% of participants reported eating unhealthily in response to stress (Mental Health Foundation, 2018). Stress not only impacts long-term health and mortality directly, but it also indirectly affects health outcomes by provoking unhealthy lifestyle behaviours, such as stress-induced eating (Leow, Jackson, Alderson, Guelfi, & Dimmock, 2018). Stress-induced eating is common, even amongst those without eating disorders, and is generally described as a harmful coping strategy (Stallman, 2020). Improving perceptions of control over risk may be vital for encouraging positive health behaviours, such as a healthy diet. However, efforts to do so may be hampered where harmful coping strategies for dealing with stress compete with the desire to eat healthily. This highlights the need for continued public health efforts to encourage the healthy and effective management of stress (NHS England, 2021).

### Informational sources

Family medical history was described by our sample as an informative source concerning potential causes of death. Though some participants did refer to the impact that familial incidents of ill health had on their own awareness of certain mortality risks, most discussions of family medical history centered on the age of death of family members. Family medical history was the most cited source of information when providing a score for perceived uncontrollable mortality risk. This supports previous analysis of data from the Health and Retirement Study which found that individual longevity predictions were more responsive to precise information (age of parental death) than imprecise information (age of parent-in-law’s death)(Chen, 2013). Participants described the potential to fixate on the age of death of family members when reflecting on their family medical history, instead of assessing which specific health risks they may be at greater risk of developing. Genetic screening and formal assessments of family medical history can be informative for determining predispositions for certain diseases (Acheson et al., 2010; Wang et al., 2012). However, emphasising the pertinence of genetic and family medical history relevant to causes of death that are broadly perceived as being uncontrollable may exacerbate determinist perceptions of control which may hinder efforts to improve certain health behaviours.

The majority of participants discussed consulting ‘*Dr Google*’ when looking for information about risks to their long-term health and safety. Some participants were keen to highlight the utility of specific websites created by the NHS and health charities for validating information found elsewhere online. However, some participants referred to Google as a primary source of health and risk-related information, rather than the means through which information sources (i.e. specific websites and user accounts) are accessed. This suggests that there are varying degrees to how discerning individuals are when selecting Google search results concerning health and risk-related information. The extent to which our sample described the internet as providing their key source of risk information highlights the importance of addressing the growing problem of health misinformation online, described by the WHO as an ‘Infodemic’ (World Health Organization, 2021a). Our sample reported the belief that health-related misinformation is prevalent on social media. This supports recent research which found that 40% of Facebook posts concerning NCDs contain health misinformation (Suarez-Lledo & Alvarez-Galvez, 2021; Waszak et al., 2018). It is noteworthy that all but one participant from our sample felt they would be unlikely to believe health misinformation online, but thought that other people are definitely susceptible to believing it. If there is a general perception that health misinformation is a ‘them, not us’ problem across the broader UK population, it may be important to bolster the reach of the UK Government’s existing guidance on identifying health misinformation in order to attempt to nullify its impact on health perceptions and behaviours (UK Government, 2021). Given the increasing importance of social media, and the internet more broadly, for providing information about long-term risks to health and safety, it is important that health communicators have the ability to ‘cut through the noise’ and are not hampered by the proliferation of trivial and misleading content (Khan, 2020; Li, Bailey, Huynh, & Chan, 2020; Ofcom, 2021; Renshaw, Mai, Dubois, Sutton, & Butts, 2021). However, it should be noted that participants’ classification of what they considered to be health misinformation was often based on their own disagreement with the information presented online. Further study is needed to verify the relationship between the perceived prevalence of health misinformation on social media, and the objective classification of health misinformation by subject experts.

Previous categorical divisions have been made between different sources of health information. For example, prevalent sources of health information have been divided into *Mass Media, Internet and Print, Support Organisations, Family and Friends, and Health Care Providers* (Blanch-Hartigan & Viswanath, 2015). In the case of mortality risks, there are clear qualitative differences in the information that is likely to be received from different sources. For example, information about the controllability of mortality risk derived from one’s family medical history is likely to contain an emotional element relevant to the death or ill health of a family member. This contrasts with the impersonal information accessed via a medical website that informs of the objective rates of a particular risk. However, given the increasing range of online health information (Rowlands et al., 2015), the lines between objective and personal may be blurred with respect to information about individual mortality risk. It is important that future studies address the changing dynamics of health and risk information online, such as the increasing variety of channels through which health messages are communicated, as well as growing consumer trust concerns. Understanding these changes will be necessary for capturing both the qualitative and quantitative differences between various sources of mortality risk-related information.

### Limitations

A limitation of this study is that participants only discussed their attitudes and beliefs about control over risk at one point in time. Both qualitative and quantitative studies of risk have typically investigated participant perceptions at a particular moment in time and may fail to capture the emergence and change of perceptions of control over time (Hawkes & Rowe, 2008). Future research may take advantage of developments in research methodologies for longitudinal qualitative interviews (alongside longitudinal quantitative studies) to provide an in depth analysis of perceptual change over time (Hermanowicz, 2013; Saldaña, 2003). This may help to explore the stability of perceptions of control and to examine how responsive these perceptions may be to external incidents of risk (i.e. the outbreak of COVID-19 and subsequent vaccine development).

Our sample was recruited via Facebook community pages which may have influenced our findings with respect to the use of different informational sources. Since all participants were social media users, our sample’s discussion of the growing prevalence of health misinformation on social media may not be representative of the attitudes of less frequent users. All interviews were conducted online with participants in their home environment. Perceptions of risk may be responsive to environmental cues, meaning that a participant’s home environment may elicit different perceptions of risk than external settings. Future studies may look to study perceptions of risk in different surroundings to further explore the role that environmental cues play in determining risk beliefs.

Finally, though participants discussed the controllability of a range of health-related causes of death, it was apparent that participants did not always distinguish between the risk of contracting a particular disease from the risk of dying from it. All discussed risks contained the potential to bring about death. However, the degree to which an individual feels they are likely to die from a specific risk, rather than it non-fatally affecting their health, may influence their beliefs about controlling the risk and subsequent health behaviours. Further research should look to unravel perceptions of direct causes of death from other health-related risks in order to better understand the complexities of perceived uncontrollable mortality risk.

## Conclusion

This interview study aimed to investigate perceptions of control over different mortality risks and asked participants to describe the sources they believe inform their perceptions of risk. Though dying from heart disease was broadly reported as being a controllable risk due to the described strong links between health and lifestyle and increased risk, the risk of dying from cancer was mostly considered to be uncontrollable. The only specific form of cancer that was discussed as being a controllable risk was lung cancer. Gender-specific cancers (notably breast cancer) were perceived as posing a significant risk to life, however discussed efforts to control the risk referred to screening and treatment adherence, not prevention. For specific cancer risks, where causal factors may not be obvious to the individual, messages that emphasise the broader links between diet, alcohol and general cancer risk may help to highlight the controllability of cancer risk through improved health behaviours. Finally, external mortality risks such as traffic accidents and the effects of air pollution were identified as potential causes of death that are generally believed to be beyond individual control. The perceived uncontrollability of these risks related to an uncertainty over specific actions that could be taken to reduce exposure to risk.

Our in-depth qualitative analysis has helped us to bridge between perceived levels of risk and information seeking behaviours to understand how perceptions of control over mortality risk may be formed. Family medical history was discussed as an informative source for perceived longevity predictions, however it was discussed less in terms of informing relatives of an increased risk of specific causes of death. Emphasising the importance of both family medical history and genetic factors may increase awareness of personalised health risk factors. However, health messages that do so must be careful not to reinforce perceptions of uncontrollability concerning specific health risks which may further disincentivise preventive health behaviours associated with the risk. Most information concerning control over risk is received from ‘*Dr Google*’, though trusted sources such as the NHS and various health charities are sometimes reported as playing a vital role in validating this information. Trust is increasingly seen as a central component of engaging with digital health information, particularly among the growing portion of society living with existing health conditions (Brown et al., 2022a; Brown et al., 2022b; Simpson et al., 2021). Given the rise in health misinformation, and the belief that it is other people not ourselves that are typically susceptible to believing misinformation online, further attempts are needed to combat this growing ‘infodemic’. This is crucial given the potential effect that health misinformation may be having on perceptions of uncontrollable mortality risk and the resulting impact on health behaviours.

## Supplementary Material

Supplemental Material
