# Supplementary material for: A qualitative study of perceptions of control over potential causes of death and the sources of information that inform perceptions of risk
Source: Health Psychol Behav Med. 2022 Jul 29;10(1):632–54. doi: 10.1080/21642850.2022.2104284 (PMC9341328; doi:10.1080/21642850.2022.2104284)
Supplement: Supplemental Material [file RHPB_A_2104284_SM6306.docx]

**Supplement**

**Item S1. Categories of mortality risk presented as discussion prompts**

*The following categories of mortality risk were provided for consideration by participants to aid in the discussion and consideration of a range of potential causes of death.*

**Public risks considered most serious and likely from the UK National Risk Register 2020 (Cabinet Office, 2020):**

1. COVID-19 or other similar outbreak of infectious disease
2. Violent attack
3. Environmental hazards (such as flooding, extreme temperatures, etc.)
4. Future effects of climate change
5. Antimicrobial Resistance
6. CBRN attack (chemical, biological, radiological or nuclear attack)

**Categories of avoidable death (Office for National Statistics, 2021):**

1. Cancer
2. Heart Disease
3. Respiratory Disease
4. Alcohol & Drugs
5. Infection
6. Accidental Injury

**Item S2. Initial Template**

1. **What types of mortality risks are perceived to be controllable/uncontrollable?**
   1. Health Risks
      1. Cancer
         1. A small degree of cancer risk is believed to be avoidable.
         2. A lot of cancer risk is believed to be unavoidable.
      2. Heart Disease
         1. Risk of heart disease is strongly linked to lifestyle, especially diet.
      3. Diabetes (type unspecified)
         1. It’s about what you eat, but familial diabetes increases your risk.
      4. Infections
         1. COVID-19
            1. If you’re going to get it, you’ll get it.
            2. You can’t control what other people do.
            3. Follow the recommended measures and you can stay safe.
            4. The pandemic has highlighted the importance of staying healthy and what you can/can’t control.
         2. General infections
            1. COVID-19 has changed the way people think about their risk of dying from general infections.

People are now more aware of the things they can do to avoid becoming infected.

People are now more aware of the things they can do to prevent infecting others.

- - 1. Less common health and mortality risks (i.e., sepsis, motor neuron disease, multiple sclerosis).
       1. There is nothing we can do to mitigate our risk of dying from some health risks.
    2. Health behaviours
       1. We are in control of our lifestyles and health behaviours.
       2. Diet is believed to be the most important factor for controlling mortality risk.
       3. We can control how susceptible we are to the effects of ill health more than our risk contracting disease.
       4. Pursuing a healthy lifestyle is balanced against competing interests.
          1. Factors relevant to quality of life (sensory pleasure, socialising, entertaining) are seen to compete with a healthy lifestyle.
          2. Pursuing a healthy lifestyle requires strong willpower.
          3. Strategies to self-manage stress may come into conflict with healthy behaviours.
  1. External Risks
     1. Traffic accidents
        1. Roads are increasingly dangerous. You can’t control other drivers.
        2. Good safety measures can reduce your risk of death.
     2. Accidents in the home
        1. Falling in the home poses a risk to life.
        2. House fires are a perceived risk for some.
     3. Pollution
        1. Air pollution (from traffic) is a live concern for many people.
        2. Our water is believed to be safe, but it could be impacting our health.
        3. Contaminants from modern farming and agricultural practices are concerning for some, though there may be little we can do to avoid them.
        4. Despite being concerned about pollution, most people do not think of these concerns in terms of their potential impact on health.
     4. Violence and attack
        1. Most people feel that risk of violence can be prevented by avoiding dangerous situations.
        2. Violent attacks that can’t be avoided are extremely rare.

1. **What are the sources of information that inform perceptions of uncontrollable mortality risk?**
   1. Sources of information
      1. Family medical history
         1. The age of death of close family members is often used to calculate individual life expectancy.
         2. Cause of death, and family illness increase awareness of specific health risks.
         3. Seeing the impact of negative health behaviours (smoking, drinking and following a poor diet) increases awareness of specific health risks.
         4. Some people look to their family medical history to feel more optimistic about their projected longevity.
      2. Healthcare Professionals
         1. Provide a trusted source of information.
         2. There are limits to the value of the information provided. Healthcare professionals typically provide a ‘one size fits all’ account of mortality risk.
      3. Traditional Media (TV, radio and print media)
         1. TV programmes about health and death can be informative and entertaining.
         2. People don’t feel traditional sources provide them with much information about risks to their life.
      4. Internet
         1. Google is generally consulted first when looking for information about risks to one’s health.
         2. Some people look to validate information by turning to trusted sources (NHS, charities, health organisations and familiar voices), some don’t.
         3. Health misinformation online
            1. Most people are aware of the prevalence of health misinformation online, particularly on social media.
            2. Widely reported by participants that they don’t feel susceptible to believing health misinformation, but other people do believe it.
      5. Information from self-monitoring of health and lifestyle
         1. Widely used to increase awareness of own health and wellness but provides little information about risks to life.
         2. Self-awareness of personal state of health is useful for identifying potential risks.
      6. Community
         1. Perceptions of the surrounding community impact feelings of safety.
         2. Familiarity with one’s surroundings may increase feelings of control.
   2. Information-seeking behaviours
      1. Most people reactively search for specific information about mortality risks in response to a particular health event.
      2. Few people proactively search for information about general health or mortality risks in response to lifestyle choices.
      3. Some people are aware that they avoid unwelcome risk information.
      4. There are some societal expectations concerning searching for information about risks to health.

**Table S1. Participant Scores for Perceived Uncontrollable Mortality Risk**

| **Participant** | **Perceived Uncontrollable Mortality Risk (0-100)** |
| --- | --- |
| 1 | 9 |
| 2 | 19 |
| 3 | 35 |
| 4 | 10 |
| 5 | 20 |
| 6 | 10 |
| 7 | 1 |
| 8 | 15 |
| 9 | 13 |
| 10 | 60 |
| 11 | Not Provided |
| 12 | 20 |
| 13 | 28 |
| 14 | Not Provided |
| 15 | 14 |
| 16 | 25 |
| 17 | 32 |
| 18 | 15 |
| 19 | 19 |
| 20 | 10 |
| 21 | 34 |
| 22 | 29 |
| 23 | 20 |
| 24 | 30 |

^Participants provided a score for their believed likelihood of living to 81 (current average UK life expectancy) provided they make the maximum effort to look after their health. This score was subtracted from 100 to provide a measure of Perceived Uncontrollable Mortality Risk (Pepper & Nettle, 2014b). A score of 0 indicates that a participant believes they have a high degree of control over their risk of death, whereas a score of 100 suggests they perceive their risk of death as being entirely uncontrollable. Two participants were unable to provide a task score due to technical difficulties during the interview. The summary of the results is as follows:^ *^N^* ^= 22,^ *^Mean^* ^= 21.3,^ *^SD^* ^= 12.6,^ *^Min^* ^= 1,^ *^Max^* ^= 60.^
